# Influence of Humidity on the Acoustic Properties of Mushroom Mycelium Films Used as Sensitive Layers for Acoustic Humidity Sensors

**DOI:** 10.3390/s20092711

**Published:** 2020-05-09

**Authors:** Iren Kuznetsova, Boris Zaitsev, Larissa Krasnopolskaya, Andrey Teplykh, Alexander Semyonov, Anastasia Avtonomova, Mayya Ziangirova, Andrey Smirnov, Vladimir Kolesov

**Affiliations:** 1 Kotelnikov Institute of Radio Engineering and Electronics of RAS, Moscow 125009, Russia; andre-smirnov-v@yandex.ru (A.S.); kvv@cplire.ru (V.K.); 2 Kotelnikov Institute of Radio Engineering and Electronics of RAS, Saratov Branch, Saratov 410019, Russia; zai-boris@yandex.ru (B.Z.); teplykhaa@mail.ru (A.T.); alex-sheih@yandex.ru (A.S.); 3 Gause Institute of New Antibiotics, Moscow 119435, Russia; lmkrasnopolska@gmail.com (L.K.); ziangirova.m@gmail.com (M.Z.); 4 Centre for Strategic Planning of Federal Medico-Biological Agency of Russia, Moscow 119121, Russia; nomova@yandex.ru

**Keywords:** mycelium film, *Pleurotus eryngii*, *Ganoderma lucidum*, BAW resonator, electrical impedance, resonant frequency, water vapor, humidity sensor

## Abstract

The influence of humidity on the density, shear elastic module, viscosity, and thickness of the mushroom *Pleurotus eryngii* and *Ganoderma lucidum* mycelium films was studied. These data were obtained by comparing the theoretical and experimental frequency dependencies of the complex electrical impedance of bulk acoustic wave (BAW) resonator loaded by mycelium film using the least-squares method. This procedure was performed for the BAW resonator with pointed films for the relative humidity range of 17%–56% at the room temperature. As a result, the changes of the density, shear elastic module, viscosity, and thickness of the films under study, due to the water vapor adsorption, were determined. It has been established that the properties of mycelium films are restored after removing from the water vapor. So, these results show the possibility of using investigated mycelium films as sensitive layers for acoustic humidity sensors.

## 1. Introduction

Humidity is a very important parameter that must be controlled in many areas of our life. The humidity sensors are used in museums, cars, “smart” houses, plants, etc. There are a lot of humidity sensors based on various physical principles (resistive [1,2], conductive [3], acoustic [4,5], capacitive [6,7], etc.). As for acoustic humidity sensors, there exists a large amount of papers, suggesting the use of film bulk acoustic resonators (FBARs) [8,9], various types of surface acoustic waves [4,5,8,9,10,11], quartz crystal microbalances (QCM) [8,12], and plate acoustic waves [13]. Most of the acoustic humidity sensors are based on the use of specific films deposited on the surface of acoustic waveguides [14]. The physical properties of such films are affected by exposure to water vapor. These, in turn, affect the acoustic wave phase velocity and/or attenuation or resonant frequency. Both organic and inorganic materials have been used as sensitive films [4,5,7,8,9,10,11,12,13,14,15]. One of the opportunities that is poorly studied is the use of films of the mycelia from higher fungi. Earlier it was experimentally stated that several mushroom mycelium extracts exhibited the high sorption sensitivity to the phenol, ammonia, formaldehyde and ethylacetate [16,17]. In this paper, we study the possibility of using other types of mushrooms as a sensitive coating for acoustoelectronic humidity sensors.

## 2. Experimental Methods

### 2.1. Preparation of Mycelium Films

#### 2.1.1. Mushroom *Pleurotus eryngii* Mycelium Film

One of the objects of this work was the culture of king oyster mushroom *Pleurotus eryngii* (D.C.) Quél. (family Pleurotaceae, class Agaricomycetes, phylum Basidiomycota). This is an edible and medicinal basidiomycete capable of synthesizing biologically active compounds [18,19]. *P. eryngii* strain 2 from the collection of the laboratory of biologically active compounds’ biosynthesis of the Gause Institute of New Antibiotics was also used. Storage and cultivation of the *P. eryngii* were carried out by the methods described previously [20]. We used the mycelium film formed by *P. eryngii* in Petri dishes (9 cm in diameter) on a potato-glucose medium. The culture was grown until complete overgrowth of Petri dishes in the dark at 25 °C. The duration of the cultivation process was 7 days.

The micromorphology of the mycelium film was studied by means of electron scanning microscopy CamScan-S2. The test material was treated with 2.5% glutaraldehyde. Then, the mycelium was washed with phosphate buffer and dehydrated by passing through ethanol solutions with increasing concentration: 30%, 50%, 70%, and 96%. Ethanol was removed and the material was treated two times with acetone. Upon completion of drying, palladium and gold were sprayed onto the material.

**Figure 1:**
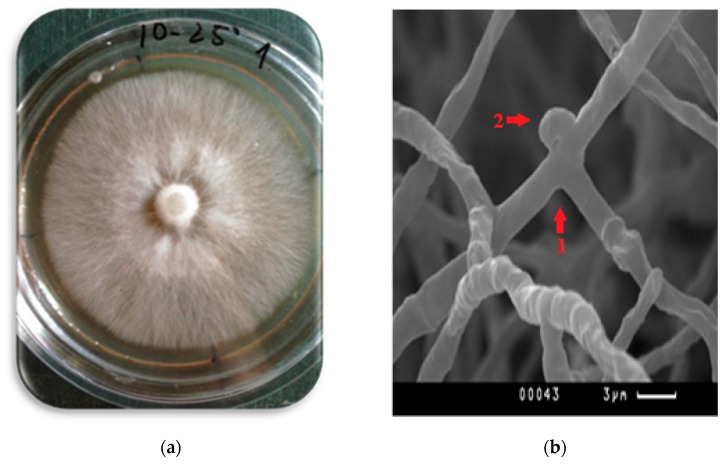
Colonies of P. eryngii (**a**) and an SEM image of *P. eryngii* mycelium (**b**): 1—branching hyphae; 2—clamp connection.

Colonies (mycelium film) of *P. eryngii* were formed by well-developed aerial mycelium consisting of radially growing branching hyphae. Colonies were white and had a felt texture; agar was not pigmented (Figure 1a). Studies of the micromorphological features of aerial mycelium showed the presence of hyphal septa and clamps on fungal hyphae (Figure 1b). Clamps are a micromorphological feature that confirms the belonging of cultures to the phylum Basidiomycota, since the formation of clamps is associated with the division of dicaryotic cells of basidiomycetes.

#### 2.1.2. Mushroom *Ganoderma lucidum* Mycelium Film

The other object of this work was the culture *Ganoderma lucidum* (Curt.) P. Karst. (phylum Basidiomycota). It is a well-known xylotrophic hymenomycete, used for medicinal purposes for over two thousand years.

Intensive studies of metabolites from *G. lucidum* fruit bodies and vegetative mycelium allowed isolate and study biologically active metabolites, especially polysaccharides and triterpenes. These compounds have immunostimulating, antitumor, hypolipidemic, antidiabetic, hepatoprotective, antiviral, anti-inflammatory, antioxidant properties.

To increase the robustness and facilitate the interpretation of our results, we used the strain of *G. lucidum*, whose morphological features, trophic needs and ability to synthesize biologically active compounds were extensively studied in our previous research [21,22,23]. The studied strain belongs to the species *G. lucidum*. This was confirmed by phylogenetic studies [24]. 

**Figure 2:**
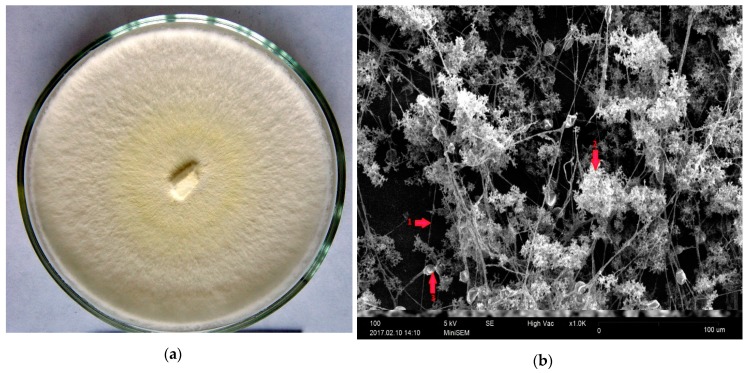
Colonies of *G. lucidum* (**a**) and SEM image of *G. lucidum* mycelium (**b**): 1—hypha; 2—coral-shaped mycelium; 3—chlamydospores.

The *G. lucidum* strain was grown on potato-glucose agar with foliferous wood sawdust for 5 days at 26 °C and then stored at 2 °C. The cultures were replanted on a fresh medium once a year. Figure 2a presents the colonies of *G. lucidum*.

The micromorphology of the intact and dried mycelium film was studied by means of the electron scanning microscopy. It has been shown that the *G. lucidum* mycelial film consists of numerous parts of the coral-shaped mycelium and an individual straight, thick and thin rarely branching hyphae bearing terminal and intercalary chlamydospores (Figure 2b). 

**Figure 3:**
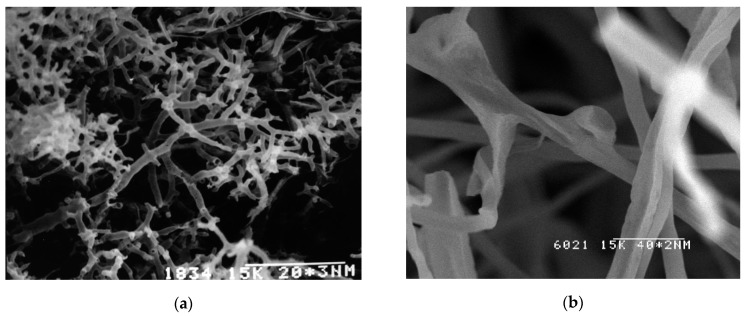
SEM images of *G. lucidum* mycelium. The coral-shaped mycelium (**a**) and clamp connection (**b**).

Figure 3 shows the high branched coral-shaped mycelium and clamp connection. 

### 2.2. BAW Resonator Loaded by Film under Study

**Figure 4:**
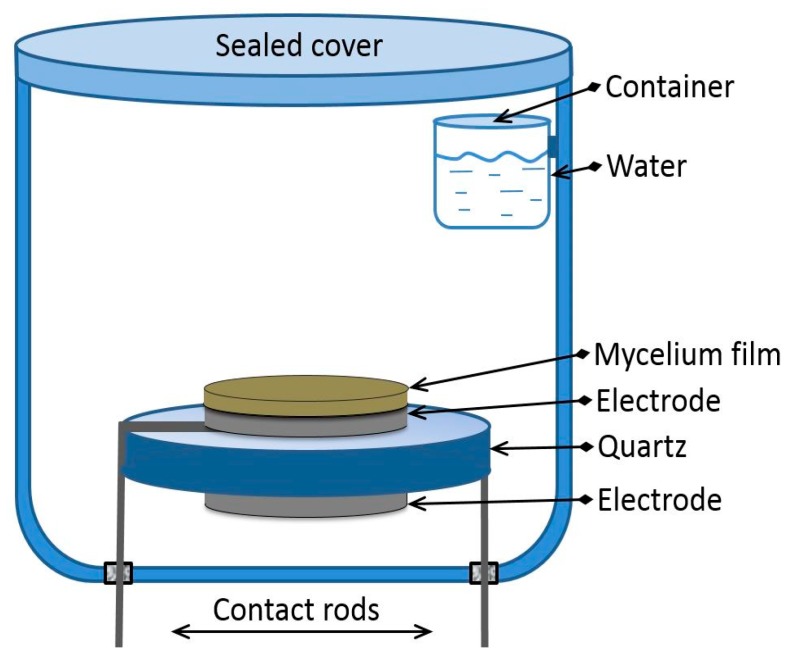
Test chamber with resonator loaded with mycelium film under study.

The experiments were performed using a standard AT-quartz resonator with the shear acoustic wave and longitudinal exciting electric field with the resonant frequency of ~4 MHz (Figure 4). The diameter of the electrodes and the thickness of the quartz plate were equal to 6 and 0.394 mm, respectively. The sample of the mycelium film with diameter of 6 mm was cut out from the original grown film in Petri dish. After that, the nutrient medium contacting with the underside of the mycelium film was mechanically removed. Then, the film was put on the electrode of the quartz resonator and glued by a wet side by uniform pressure and smoothing. After that the resonator with film was kept in air for 24 h. This method allowed for obtaining the mycelium films with a thickness of about 20 μm. Such films of mycelium possessed sufficient mechanical strength and were used in our experiments.

### 2.3. Test Chamber

The special glass chamber used for investigation of the mycelium film properties at different values of humidity is presented in Figure 4. The volumes of chamber and container for water were equal to ~100 ml and ~2 ml, respectively. The chamber was equipped by the humidity and temperature resistive sensors of the meter MAX-MIN Thermo Hygro (Shenzhen Shining Electric Technolodgy Co., Shenzhen, China). The AT-quartz resonator was placed inside the chamber and connected with the contact rods hermetically fixed to the bottom of the chamber. The chamber was hermetically closed by the cover with a Teflon sealing. This system allowed for smooth increasing the relative humidity from 17% to ~60% for 1 h. The measurements were carried out at the room temperature (24 °C) and a normal atmosphere pressure of 760 mm of mercury. A special attention was paid to the electrical quality of the contacts between the resonator and the measuring set up. This chamber was used for investigation of the influence of the water vapors on the mechanical properties of the mycelium films under study.

### 2.4. Method of the Measurement of the Physical Characteristics of the Films under Study

**Figure 5:**
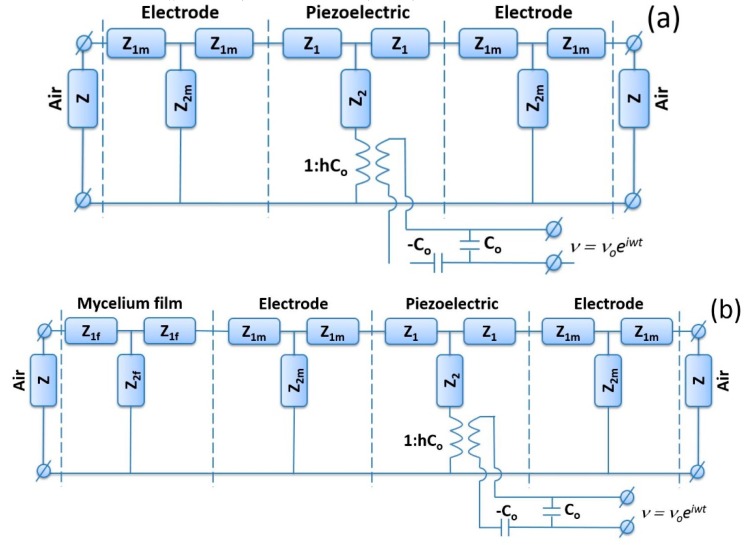
Mason’s equivalent circuits for the resonator with electrodes (**a**) without and (**b**) with the mycelium film under study.

At first, we measured the frequency dependencies of the real (Re) and imaginary (Im) parts of the complex electrical impedance for the quartz resonator without film by means of a precision LCR meter (4285A, Agilent Technologies, Santa Clara, CA, USA). The Mason’s equivalent circuit of a resonator that has been exploited to calculate the theoretical frequency dependencies of the resonator electrical impedance is shown in Figure 5a [17,25]. This circuit considers the parameters of the quartz plate and electrodes because their thickness is commensurable with the thickness of the films under study. 

The mechanical impedances *z_1_*, *z_2_*, *z_1m_*, and *z_2m_* in the equivalent scheme presented in Figure 5a were presented in the following form [17,25]:

*z_1_* = *iZS* tg(*kd*/2); *z_2_* = −*iZS*/sin(*kd*/2); (1)

*z_1m_* = *iZ _m_ S* tg(*k_m_d_m_*/2); *z_2m_* = −*iZ_m_ S*/sin(*k_m_d_m_*/2) (2)

where *Z* = (*C_66_ρ*)^1/2^ and *Z_m_* = (*C_66_^m^ρ_m_*)^1/2^ are the specific acoustic impedances of the resonator and electrode’s materials, respectively. *С_66_*, *C_66_^m^*, *ρ*, and *ρ_m_* are their shear elastic constants and densities, respectively. *k* = *ω*/*v* and *k_m_* = *ω*/*v_m_* are the corresponding wave numbers. *ω*, *v*, and *v_m_* are the angular frequency and phase velocities of the wave in the AT-quartz and electrodes. *S*, *d*, and *d_m_* are the electrode area and the thicknesses of resonator and electrode, respectively. *i* is the imaginary unit. Index *m* is related to the electrodes. 

The equivalent circuit consists of an electromechanical transformer responsible for the mutual transformation of electrical and mechanical oscillations [25]. A primary electrical winding is in contact with elements *C_0_* and −*C_0_*, and the secondary mechanical one is in contact with resistors *z_1_* and *z_2_*. The transformer ratio *N* is given by *N* = *hC_0_*, where *h* = *e_25_*/*ε_11_* and *e_25_*, *ε_11_* are the piezoelectric constant and permittivity of the quartz, respectively. *C_0_* = *ε_11_S*/*d* is the electric capacity. The effective viscosity *η_66_* of the resonator material was considered as the main source of losses, including the mechanical and electrical ones. In this case, the velocities and the specific acoustic impedances of the resonator and electrodes may be written as follows [25]:

*v* = {(*C_66_* + *e_25_*^2^/*ε_11_* + *iωη_66_*)/*ρ*}^1/2^, Z = {(*C_66_* + *e_25_*^2^/*ε_11_* + *iωη_66_*)*ρ*}^1/2^, (3)

*v_m_* = {*C_66_^m^*/*ρ_m_*}^1/2^, *Z_m_* = {*C_66_^m^ρ_m_*}^1/2^. (4)

The Kirchhoff’s equations were applied with the circuit shown in Figure 5a to calculate the frequency dependencies of the real and imaginary parts of the resonator electrical impedance for its particular material constants (C_66_, C_66_^m^, e_25_, ε_11_, η_66_, d_m_) [26]. We varied these material constants in the given limits and exploited the least-squares method to find a set of abovementioned material constants that offered the closer agreement of theoretical and experimental frequency dependencies of the complex electrical impedance. 

Then, the resonator electrode was covered by the mycelium film. Through 24 h, the loaded resonator was set in the gas chamber (Figure 4), and, by means of the LCR meter, (4285A, Agilent Technologies, Santa Clara, CA, USA) the frequency dependencies of the real and imaginary parts of the complex electrical impedance of the loaded resonator were measured. By using the equivalent circuit presented in Figure 5b and the Kirchhoff’s equations, the theoretical dependencies of the real and imaginary parts of electrical impedance of the loaded resonator were calculated. The acoustical impedances *z_1f_* and *z_2f_* of the mycelium film were computed as:

*z_1f_* = *iZ _f_ S* tg(*k_f_d_f_*/2); *z_2f_* = −*iZ_f_ S*/sin(*k_f_d_f_*/2), (5)

where *Z_f_* = (*C_66_^f^ρ_f_*)^1/2^ is the specific acoustic impedance of the film under study. *C_66_^f^* and *ρ_f_* are shear elastic constant and density of the film, respectively. *k_f_* = *ω*/*v_f_* is the wave number. *v_f_* is the phase velocity of the acoustic wave in the film. *d_f_* is the thickness of the film. Index *f* is related to the mycelium investigated film. The phase velocity of wave and the specific mechanical impedance of the film were determined in accordance with the following expressions:

*v_f_* = {(*C_66_^f^* + *iωη_66_^f^*)/*ρ_f_*}^1/2^, *Z_f_* = {(*C_66_^f^* + *iωη_66_^f^*)*ρ_f_*}^1/2^. (6)

**Figure 6:**
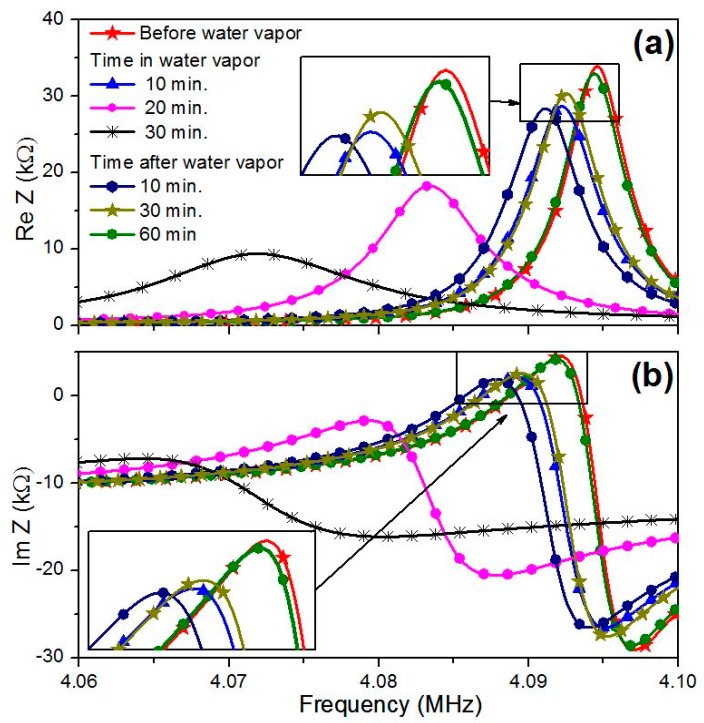
Frequency dependencies of (**a**) the real and (**b**) imaginary parts of the complex electrical impedance of the AT-quartz resonator loaded by the mushroom *P. eryngii* mycelium film.

**Figure 7:**
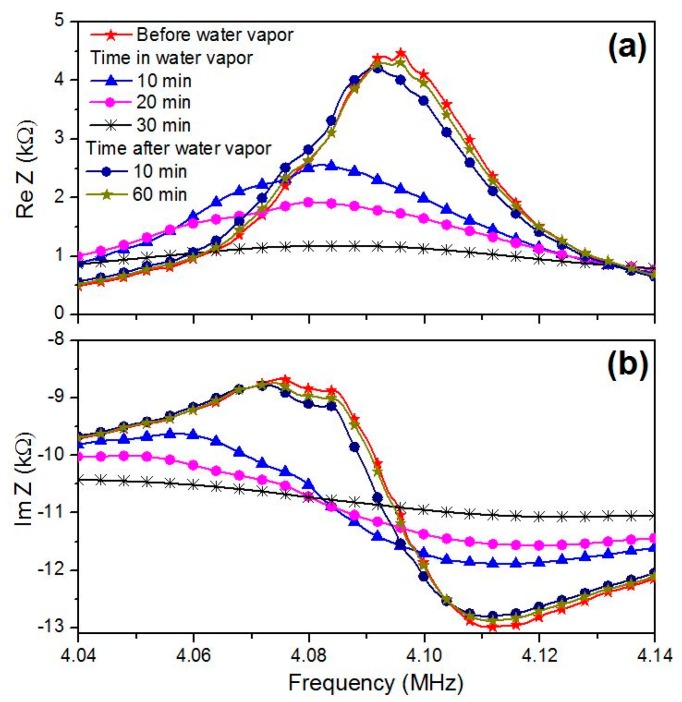
Frequency dependencies of (**a**) the real and (**b**) imaginary parts of the complex electrical impedance of the AT-quartz resonator loaded by the mushroom *G. lucidum* mycelium film.

In analysis, we used the values of the material constants of quartz (*C_66_*, *e_25_*, *ε_11_*, *η_66_*) and the parameters of electrodes (*C_66_^m^*, *ρ_m_*, *d_m_*) that were determined on a previous step. The material constants *C_66_^f^*, *ρ_f_*, *η_66_^f^* and thickness *d_f_* of mycelium film also were considered in calculations. By changing the values of material constants and the thickness of mycelium film in the given ranges, we found set of the film parameters *C_66_^f^*, *ρ_f_*, *η_66_^f^*, and *d_f_* with the help of the least-squares method. Curves presented by solid lines in Figure 6 and Figure 7 correspond to the calculated frequency dependencies of the real (a) and imaginary (b) parts of the electrical impedance for the resonator loaded by electrodes and mycelium films under study, which are most adjusted to the experiment (points in Figure 6 and Figure 7). 

Figure 6 and Figure 7 show that, with an increase in humidity, the values of the real and imaginary parts of the electrical impedance and the resonant frequency decrease.

The mass m_f_ of the studied films was also determined after “fitting” by using known film area *S* and obtained values of density *ρ_f_* and thickness *d_f_*:

*m_f_* = *S* × *d_f_* × *ρ_f_*. (7)

Then, the chamber was filled with water vapors, and a bulk acoustic wave (BAW) resonator with mycelium film was kept inside the chamber for 10 min. After this, the frequency dependencies of the real and imaginary parts of the electrical impedance were measured and the parameters of the film were calculated again. This procedure was also repeated at 20, 30 and 60 min. Then, the cover was opened and the water container was removed from the chamber. After this, the aforementioned dependencies were measured in air at 10, 20, 30 and 60 min. Correspondingly, each time we calculated the thickness and material constants of the film without fail. The change in the parameters of mycelium films in the presence of the water vapor allowed us to make conclusion about the sorption properties of the films under study. 

The humidity meter MAX-MIN Thermo Hygro (Shenzhen Shining Electric Technolodgy Co., Shenzhen, China) allowed us to construct the time dependence of the humidity (RH%), which is presented in Table 1.

Frequency step of the measurement of frequency dependencies of the impedance was equal to 200 Hz and the accuracy of the impedance measurement was 1%. 

## 3. Results and Discussion

As the result of the performed experiments and corresponding calculations, the values of the density (*ρ_f_*), shear elastic module (*C^f^_66_*), effective viscosity *(η_66_^f^*), thickness (*d_f_*), and mass (*m_f_*) of the mushroom *P. eryngii* and *G. lucidum* mycelium films at different values of humidity were obtained. These data are presented in Table 2 and Table 3, respectively.

The analysis of the obtained results has shown that the increase in humidity leads to decreasing the resonant frequency and the values of the real and imaginary parts of electrical impedance. This trend is the same as presented earlier in [17]. Additionally, it has been found that the thickness, viscosity, density and mass of the films under study increase with an increase in humidity and return to the initial values after 60 min in air. The shear elasticity module shows the opposite behavior. It reduces due to the humidity increase and fully recovers when humidity takes the initial value (17%). This may be explained by the penetration of the water molecules inside the film that leads to the film swelling and decreases its elasticity. After removing from the water vapor, the film dried out during some time and its physical properties returned to the initial values.

It should also be noted that the electrical impedance of a resonator loaded with a *G. lucidum* mycelial film is almost an order of magnitude smaller than for the *P. eryngii* film (Figure 6 and Figure 7). This may be connected with the structural features of various types of the mycelium, although the growth environment was the same for them. An analysis of the results also shows that the water vapor has a much stronger effect on the thickness, elastic modulus, viscosity and mass of the *G. lucidum* film in comparison with the *P. eryngii* film. For example, the relative changes in thickness, shear elastic modulus, the coefficient of viscosity, and mass for *G. lucidum* film were 9.8%, 38%, 21.6% and 15.7%, respectively. At that for *P. eryngii* film, these values were equal to 3.6%, 19%, 37.5%, and 9%. One can also see that the density of the *G. lucidum* films is higher than one of the *P. eryngii* films. The noted differences between the properties of the mycelial films of two species of the basidiomycetes are consistent with the results of mycological studies. It is known that after the colonization of a dense nutrient medium with the fungal hyphae, the properties of *P. eryngii* aerial mycelium (color, height) remain constant. At the end of the mycelium radial growth, colonies of *G. lucidum* undergo significant changes. They become denser, the height of the aerial mycelium decreases, the yellow or brown pigment appears and the viscosity of fruit body increases. As a result, a morphostructure is formed, which performs the protective functions [27]. The results obtained allow to us suggest that an important stage in the formation of the protective mycelium film of *G. lucidum* is a significant loss of water by the mycelial cells. Apparently, this process is not passive, but active, since the nutrient medium at this time is saturated with water.

We have compared our data with the analogous data presented in [15]. The authors studied the influence of RH on the mechanical properties of the acetate chitosan film. It was shown that the increase in the RH from 20% to 60% practically did not change the elastic constants *C_11_* and *C_44_* of the chitosan film and led to the increase in the viscosity factor by 26.6%. In the present work, the growth of the relative humidity in the range 17%–56% changed the elasticity and viscosity coefficients by 38% and 21.6% for the *G. lucidum* film and by 19% and 37.5% for the *P. eryngii* film, respectively. Such combinations led to a stronger effect of the humidity on the parameters of the resonator on quartz compared with the piezoceramic (Ba0.24Pb0.75Sr0.01(Ti0.47Zr0.53)O3) resonator described in [15].

## 4. Conclusions

The influence of water humidity on the density, shear elastic module, viscosity coefficient, and thickness of mushroom *P. eryngii* and *G. lucidum* mycelium films was studied. The *P. eryngii* and *G. lucidum* strains used in the work were taken from the collection of the laboratory of biologically active compounds’ biosynthesis of the Gause Institute of New Antibiotics. The pointed data were obtained by the comparison of the theoretical and experimental frequency dependencies of the complex electrical impedance of the bulk acoustic wave resonator loaded by mycelium film using the least-squares method. It has been found that the values of the density and shear elastic module of the investigated films are equal 1100 kg/m^3^ and 0.69 GPa for *P. eryngii* and 1300 kg/m^3^ and 0.28 GPa for *G. lucidum* in air. The measurements and calculations were carried out in the relative humidity range of 17%−56% at the room temperature. We determined the changes in the density, shear elastic module, viscosity, and thickness of the film under study due to the water vapor adsorption. The obtained results have shown that, with an increase in humidity, the thickness, viscosity, and density of the films under study increase, whereas the shear elastic module decreases. It has also been found that the properties of the mycelium films fully recover after being removed from the water vapor. Overall, the results obtained show the potential of using the investigated mycelium film for the development of acoustic humidity sensors.

## Figures and Tables

**Table 1:** Time dependence of the humidity (RH%) obtained in experiment.

Moment of Measurement Time	0 min in air	10 min in vapor	20 min in vapor	30 min in vapor	10 min out of vapor	20 min out of vapor	30 min out of vapor	60 min out of vapor
RH%	17	40	48	56	40	28	20	17

**Table 2:** Parameters of the mushroom *P. eryngii* mycelium film at different values of humidity.

Time	RH%	*d_f_*, μm	*C^f^_66_*, GPa	*η_66_^f^*, Pa × s	*ρ^f^*, kg/m^3^	*m_f_*, μg
0 min in air	17	22.88 ± 0.01	0.69 ± 0.01	2.5 ± 0.1	1100 ± 10	0.711
10 min in vapor	40	22.86 ± 0.01	0.67 ± 0.01	2.6 ± 0.1	1110 ± 10	0.717
20 min in vapor	48	22.86 ± 0.01	0.60 ± 0.01	2.7 ± 0.1	1150 ± 10	0.743
30 min in vapor	56	23.32 ± 0.01	0.56 ± 0.01	4.0 ± 0.1	1190 ± 10	0.784
10 min after vapor	40	22.76 ± 0.01	0.60 ± 0.01	2.1 ± 0.1	1100 ± 10	0.707
20 min after vapor	28	22.68 ± 0.01	0.66 ± 0.01	2.5 ± 0.1	1120 ± 10	0.717
30 min after vapor	20	22.91 ± 0.01	0.68 ± 0.01	2.6 ± 0.1	1110 ± 10	0.718
60 min after vapor	17	22.9 ± 0.01	0.69 ± 0.01	2.6 ± 0.1	1100 ± 10	0.718

**Table 3:** Parameters of the mushroom *G. lucidum* mycelium film at different values of humidity.

Time	RH%	*d_f_*, μm	*C^f^_66_*, GPa	*η_66_^f^*, Pa × s	*ρ^f^*, kg/m^3^	*m_f_*, μg
0 min in air	17	17.5 ± 0.1	0.28 ± 0.01	4.0 ± 0.1	1300 ± 10	0.642
10 min in vapor	40	17.6 ± 0.1	0.24 ± 0.01	4.7 ± 0.1	1370 ± 10	0.681
20 min in vapor	48	17.8 ± 0.1	0.19 ± 0.01	4.7 ± 0.1	1400 ± 10	0.704
30 min in vapor	56	19.4 ± 0.1	0.17 ± 0.01	5.1 ± 0.1	1390 ± 10	0.762
10 min after vapor	40	17.3 ± 0.1	0.26 ± 0.01	3.4 ± 0.1	1300 ± 10	0.635
60 min after vapor	17	17.3 ± 0.1	0.27 ± 0.01	3.8 ± 0.1	1300 ± 10	0.635

## References

[1] Cho N.-B., Lim T.-H., Jeon Y.-M., Gong M.-S. (2008). Inkjet printing of polymeric resistance humidity sensor using UV-curable electrolyte inks. Macromol. Res..

[2] Pustelny T., Setkiewicz M., Drewniak S., Maciak E., Stolarczyk A., Procek M., Urbanczyk M., Gut K., Opilski Z., Pasternak I. (2012). The influence of humidity on the resistance structures with graphene sensor layer. Acta Phys. Polon. A.

[3] Barkauskas J. (1997). Investigation of conductometric humidity sensors. Talanta.

[4] Caliendo C., Verona E., Anisimkin V.I. (1997). Surface acoustic wave humidity sensors: A comparison between different types of sensitive membrane. Smart Mater. Struct..

[5] Penza M., Anisimkin V.I. (1999). Surface acoustic wave humidity sensor using polyvinyl-alcohol film. Sens. Actuators A.

[6] Narimani K., Nayeri F.D., Kolahdouz M., Ebrahimi P. (2016). Fabrication, modeling and simulation of high sensitivity capacitive humidity sensors based on ZnO nanorods. Sens. Act. B Chem..

[7] Bi H., Yin K., Ji J., Wan S., Sun L., Terrones M., Dresselhaus M.S. (2013). Ultrahigh humidity sensitivity of graphene oxide. Sci. Rep..

[8] Mujahid A., Afzal A., Dickert F.L. (2019). An Overview of High Frequency Acoustic Sensors-QCMs, SAWs and FBARs-Chemical and Biochemical Applications. Sensors.

[9] Liu J., Zhao Z., Fang Z., Liu Z., Zhu Y., Du L. (2020). High-performance FBAR humidity sensor based on the PI film as the multifunctional layer. Sens. Act. B Chem..

[10] Su Y., Li C., Li M., Li H., Xu S., Qian L., Yang B. (2020). Surface acoustic wave humidity sensor based on three-dimensional architecture graphene/PVA/SiO2 and its application for respiration monitoring. Sens. Act. B Chem..

[11] Kuznetsova I.E., Anisimkin V.I., Kolesov V.V., Kashin V.V., Osipenko V.A., Gubin S.P., Tkachev S.V., Verona E., Sun S., Kuznetsova A.S. (2018). Sezawa wave acoustic humidity sensor based on graphene oxide sensitive film with enhanced sensitivity. Sens. Act. B Chem..

[12] Zheng X., Fan R., Li C., Yang X., Li H., Lin J., Zhou X., Lv R. (2019). A fast-response and highly linear humidity sensor based on quartz crystal microbalance. Sens. Act. B Chem..

[13] Kuznetsova I.E., Anisimkin V.I., Gubin S.P., Tkachev S.V., Kolesov V.V., Kashin V.V., Zaitsev B.D., Shikhabudinov A.M., Verona E., Sun S. (2017). Super high sensitive plate acoustic wave humidity sensor based on graphene oxide film. Ultrasonics.

[14] Balantine D.S., White R.M., Martin S.J., Ricco A.J., Frye G.C., Zellers E.T., Wohltjen H. (1997). Acoustic Wave Sensors: Theory, Design and Physico-Chemical Applications.

[15] Zaitsev B.D., Teplykh A.A., Fedorov F.S., Grebenko A.K., Nasibulin A.G., Semyonov A.P., Borodina I.A. (2020). Evaluation of elastic properties and conductivity of chitosan acetate films in ammonia and water vapors using acoustic resonators. Sensors.

[16] Silina Y.E., Kuchmenko T.A., Korenman Y.I., Tsivileva O.M., Nikitina V.E. (2005). Use of a complete factorial experiment for designing a gas sensor based on extracts of *Pleurotus ostreatus* mycelium mushroom. J. Analyt. Chem..

[17] Kuznetsova I.E., Zaitsev B.D., Shikhabudinov A.M., Tsivileva O.M., Pankratov A.N., Verona E. (2017). Acousto-electronic gas sensor based on mushroom mycelium extracts. Sens. Act. B Chem..

[18] Wasser S.P. (2014). Medicinal mushroom science: Current perspectives, advances, evidences, and challenges. Biomed. J..

[19] Trenin A.S., Bychkova O.P., Tsvigun E.A., Dzhavakhyan B.R., Tyurin A.P., Krasnopolskaya L.M. (2015). Obtaining sterol biosynthesis’ inhibitors in the culture of basidiomycete *Pleurotus Eryngii*. Uspekhi Med. Mikol..

[20] Krasnopolskaya L.M., Leontieva M.I., Avtonomova A.V., Isakova E.B., Belitskii I.V., Bukhman V.M., Usov A.I. (2008). Antitumor properties of submerged cultivated biomass and extracts of medicinal mushrooms of genus HYPSIZYGUS Singer (AGARICOMYCETIDEAE). Intern. J. Med. Mushrooms.

[21] Avtonomova A.V., Krasnopol’skaia L.M., Maksimov V.N. (2006). Optimization of nutrient medium for submerged cultivation of Ganoderma lucidum (Curt.: Fr.) P. Karst. Microbiology.

[22] Evsenko M.S., Shashkov A.S., Avtonomova A.V., Krasnopolskaya L.M., Usov A.I. (2009). Polysaccharides of basidiomycetes. alkali-soluble polysaccharides from the mycelium of white rot fungus Ganoderma lucidum (Curt.: Fr.) P. Karst. Biochemistry (Mosc.).

[23] Krasnopol’skaya L.M., Yarina M.S., Avtonomova A.V., Usov A.I., Isakova E.B., Bukchman V.M. (2015). Antitumor Activity of Polysaccharides from Ganoderma lucidum Mycelium: In vivo Comparative Study. Antibiot. Chemoterapy (Mosc.).

[24] Yarina M. , Krasnopolskaya  L., Usov  A., Marakhonov A. Ganoderma lucidum and Ganoderma resinaceum strains producing antitumor alkali-solublepolysaccharide xylomannan. Proceedings of the XXXVII Annual Meeting of the European Culture Collections Organization.

[25] Dieulesaint E., Royer D. (1980). Elastic Waves in Solids: Applications to Signal Processing.

[26] Kuznetsova I.E., Zaitsev B.D., Shikhabudinov A.M. (2010). Elastic and viscosity properties of Nanocomposite Films Based on Low-Density Polyethylene. IEEE Trans. Ultras. Ferroel. Freq. Contr..

[27] Vetchinkina E.P., Nikitina V.E. (2017). Effect of the cold stress on the fruiting body production by the medicinal basidiomycetes in submerged and solid-phase culture. J. Stress Physiol. Biochem..

